# Trends and outcomes of fertility preservation for girls, adolescents and young adults with Turner syndrome: A prospective cohort study

**DOI:** 10.3389/fendo.2023.1135249

**Published:** 2023-03-03

**Authors:** Kenny A. Rodriguez-Wallberg, Fotios Sergouniotis, Hanna P. Nilsson, Frida E. Lundberg

**Affiliations:** ^1^ Department of Oncology-Pathology, Laboratory of Translational Fertility Preservation, Karolinska Institutet, Stockholm, Sweden; ^2^ Department of Reproductive Medicine, Division of Gynecology and Reproduction, Karolinska University Hospital, Stockholm, Sweden; ^3^ Department of Medical Epidemiology and Biostatistics, Karolinska Institute, Stockholm, Sweden

**Keywords:** fertility preservation, Turner syndrome, fertility, ovarian tissue cryopreservation, pre-pubertal girls, adolescents

## Abstract

**Background:**

In Scandinavian countries, programs for fertility preservation (FP) are offered free of charge at tertiary-care university hospitals to all patients facing infertility risks due to malignant diagnoses or benign conditions. In this prospective study we aimed to investigate trends and outcomes of FP indicated by a diagnosis of Turner syndrome.

**Methods:**

Prospective cohort study of patients with Turner karyotype receiving fertility preservation counselling at the Karolinska University Hospital between 1 January 1999 and 31 December 2021.

**Results:**

The cohort included 100 women and girls that received counselling, whereof 27% were prepubertal girls, 59% were adolescents and 14% of adult age. Before 2006 all patients were referred for fertility counselling at the time of Turner diagnosis. Based on updated guidelines, mainly patients who showed signs of puberty were referred after 2006. As a result, spontaneous menarche was more common in the later period. In total, 39% of the cohort had monosomal karyotype (45X), 20% had 45X/46XX or 45X/47XXX mosaicisms and 36% had an X-chromosomal structural anomaly. Ovarian tissue cryopreservation was planned for 73% of all patients, and oocyte cryopreservation following gonadotropin stimulation was planned for 10% of the patients. Follicles were present in 25% of all biopsies analyzed. Adolescents were more likely to have follicles present (30%) than prepubertal girls (16%) or adult women (17%). The ten patients that underwent gonadotropin stimulation for oocyte cryopreservation underwent a total of 15 cycles and eight patients successfully preserved oocytes. In total, 26% of the cohort has undergone fertility treatment or expressed further interest in fertility preservation. Six women have given birth using donated oocytes and three following spontaneous conception. Two women have undergone re-transplantation of cryopreserved ovarian tissue, without regaining ovarian function, and none of the women that have cryopreserved oocytes has returned to use them.

**Conclusion:**

Fertility counselling for girls with Turner syndrome should ideally be offered at onset of spontaneous puberty to improve the chances of fertility preservation. Since the girls and women in this cohort are still young, the return rate and utilization of the preserved tissue and oocytes is expected to increase with time.

**Clinical Trial Registration:**

ClinicalTrials.gov, identifier NTC04602962.

## Introduction

Turner syndrome is the most common sex chromosome abnormality in women, with a prevalence of 1/2500. Diagnosis is most common in early childhood or adolescence, but in some cases the syndrome is diagnosed later in life, often related to an infertility work-up. The syndrome has a wide phenotypic spectrum caused by complete or partial absence of an X chromosome. Due to the range of possible abnormalities, including mosaicisms, Turner syndrome does not always present phenotypically and may remain undiagnosed throughout life (1). However, as widely acknowledged, Turner syndrome has a direct negative effect on fertility and most girls diagnosed with Turner syndrome will not undergo spontaneous puberty. It is estimated that approximately 20% of the girls diagnosed with Turner syndrome will spontaneously present initial puberty development, usually breast development, but only 16% of the girls will proceed to menarche (2). The likelihood to present with clinically evident ovarian function is higher in patients with X-chromosome mosaicisms (45X/46XX, 45X/47XXX) than in those having other X-chromosome variants (46X with ring chromosome, 46X with deletion, 46X with isochromosome, or 45X monosomy) (3). Girls with diagnosed Turner syndrome who show signs of ovarian function in childhood and early adolescence most often develop premature ovarian failure around the time of puberty due to rapid atresia of the follicles (4). It is currently recommended to offer reproductive counselling when a Turner diagnosis is confirmed and also to perform a careful cardiac evaluation to exclude comorbidities (5–7).

In women with Turner syndrome cardiac features may contraindicate a future pregnancy. Congenital heart abnormalities and aortic dilatation, which are strongly associated with life-threatening aortic dissection, are more common in Turner patients, where the incidence of maternal and postpartum death has been reported as high as 2% (8–10). If congenital heart abnormalities or acquired aortic dilation is present in women with Turner syndrome, medical assistance to achieve pregnancy is not recommended and other options for parenthood should be advised (5). While data are still heterogeneous, retrospective studies have indicated that with adherence to health care guidelines pregnancies can proceed without increase in cardiovascular complications (8–14).

In Sweden, a National Healthcare Program for patients with Turner diagnosis has been established since 1994, updated subsequently in 2000 and 2013, and is currently known as the Swedish Turner Academy. The program includes recommendations on how girls and women with Turner syndrome should be followed-up throughout life by multidisciplinary teams at the Turner centers established at all university hospitals (15). Since 2013, the program recommends that discussions regarding reproductive options should be initiated soon after diagnosis, and routinely revisited at the time of transition from pediatric to adult healthcare. In all patients that receive a Turner diagnosis irrespective of age, a thorough cardiac evaluation is performed with echocardiography and/or magnetic resonance imaging. This way the cardiovascular status is known before fertility preservation discussions would take place (5).

In order to preserve fertility in patients with Turner syndrome, fertility preservation (FP) should be offered at an early age, before oocyte depletion (6, 16, 17). However, there are no reliable methods to predict the progress of atresia, nor can it be determined if the follicles in pre-pubertal girls are functional or not. This makes routine implementation of FP difficult in patients with Turner syndrome. In most FP programs it is established that if a girl reaches spontaneous menarche, ovarian stimulation and collection of oocytes can be considered (18, 19). In young pre-pubertal girls, it is more difficult to establish an optimal method for FP. A previous Swedish study reporting on ovarian biopsies and ovarian tissue cryopreservation in Turner patients revealed that the probability of identifying ovarian follicles in the biopsies increased if the Turner karyotype showed a mosaicism, if the girls had developed spontaneous puberty, and if the serum Follicle-Stimulating Hormone (FSH) and Anti Müllerian Hormone (AMH) concentrations were normal for age (8). As shown in that study, biopsies were feasible in 47 of 57 girls and follicles were identified in 15 cases. Finding follicles in the ovarian biopsies supports future fertility treatment, e.g., by re-transplantation of the tissue, or, if ovarian function is evident during adolescence or early adulthood, by ovarian stimulation as a complementary option to girls who have previously cryopreserved ovarian tissue (16). A reason to offer ovarian stimulation is the high efficacy of vitrified oocytes, while re-transplantation of ovarian tissue from women and girls with a reduced ovarian reserve at time of cryopreservation is expected to have low success rate.

At Karolinska University Hospital in Stockholm, fertility counselling and FP have been offered to girls and women with the Turner syndrome as part of a clinical study at the Karolinska University Hospital in Stockholm (3), and the center has also been the national reference center for fertility preservation for children. In this study we present treatment outcomes and long-term follow-up of a large cohort of women and girls with Turner syndrome.

## Material and methods

The study cohort included all girls and women presenting with Turner syndrome who were referred for fertility preservation counselling at the Karolinska University Hospital, Section of Reproductive Medicine, in Stockholm, Sweden between 1 January 1999 and 31 December 2021. Data on referral, clinical characteristics and utilization of cryopreserved oocytes and tissues have been collected prospectively.

### Counselling of girls and teenagers

According to the recommendation of the Swedish Turner multidisciplinary program, adolescent girls who present with spontaneous start of puberty should be referred for appropriate counselling on fertility preservation, and if possible, individualized fertility preservation (15). Nevertheless, no girls referred to the clinic have been excluded from the study, independently of age or pubertal development. Over the years, women and adolescent girls have been predominantly counselled towards clinically established methods. However, the methods for fertility preservation have improved over time as have the methods to determine the female ovarian reserve. Routine hormonal measurements started to be implemented at our center around 2006 using validated clinical methods at Karolinska University Laboratory. Earlier measurements were executed at either the Research Laboratory for Women’s Health, Karolinska Institutet (FSH, LH, AMH) or the Central Laboratory for Clinical Chemistry, Karolinska University Hospital (FSH) as previously described by Borgström et al., 2009 (3). As most patients before 2006 were referred for fertility preservation regardless of hormonal status, we have divided the cohort over time before and after 2006.

In most cases, counselling was provided to the patients and their families by a pediatrician and also a reproductive medicine specialist. Written age‐adapted information, in two versions (for children and adolescents) was provided. Parents of minors were asked to sign an informed consent form; when teenagers were counselled and they assented, both the parents and the child signed the form. Counselling also included information on alternatives to becoming a parent, such as using egg donation or adoption. The possibility to undergo fertility preservation at a later stage was also discussed, in case the patients elected not to undergo FP at time of referral (20).

At the time of counselling, if deemed appropriate and if the patients agreed to these examinations, ovarian reserve was evaluated by counting antral follicles through transvaginal ultrasound and by measuring serum concentrations of anti-Müllerian hormone. In several cases the results of the exams were also used to evaluate the long-term benefit of undergoing FP.

Girls and teenagers were most often counselled to laparoscopic retrieval of ovarian tissue for cryopreservation, where the need of future re‐transplantation through additional surgeries in order to regain tissue functionality and fertility was explained. Patients were also informed on the possible advances in methods for *in vitro* tissue culturing of follicles to mature oocytes. The amount of ovarian tissue retrieved was individualized in all cases and discussed with the patients. After 2009 stimulation for oocyte cryopreservation through vitrification was also offered as an option to adolescent patients, if the girls had developed puberty and proceeded through menarche and wished to undergo the procedure, which includes monitoring with transvaginal ultrasound examinations and transvaginal follicular aspiration (19). The expected efficacy of the FP methods according to the current state of knowledge was explained.

Ethical approval for the study was granted by the Ethical Review Board of Karolinska University Hospital (Dnr 427/03) and the Regional Ethics Committee of Stockholm (Dnr 2011/1158-31/2, 2014/470-32, 2016/2530-32 and 2018/2255-32). Written informed consent to participate in this study was provided by the participants, or by the participants’ legal guardian/next of kin.

## Results

In total, 100 patients with Turner karyotype were counselled for fertility preservation between 1999 and 2021. The majority (59%) of the patients were between 13 and 17 years of age, 27% were prepubertal and 14% were adults. More than half (65%) the patients were referred for fertility counselling between 1999-2005 (**Table 1**).

**Table 1:** Description of the cohort by calendar period of referral.

	1999-2005 N=65	2006-2021 N=35	All patients N=100
Age - Mean ± SD	13.8 ± 0.35	14.5 ± 0.84	14.0 ± 0.4
Age group			
Girls (1-12y)	21 (32%)	6 (17%)	27 (27%)
Adolescents (13-17y)	37 (57%)	22 (63%)	59 (59%)
Women (18-27y)	7 (11%)	7 (20%)	14 (14%)
Turner Syndrome karyotype			
Monosomy 45X	32 (49%)	7 (20%)	39 (39%)
Mosaic 45X/46XX, 45X/47XXX	8 (12%)	12 (34%)	20 (20%)
X-chromosome structural anomaly^a^	23 (35%)	23 (35%)	36 (36%)
Missing	2 (3%)	3 (9%)	5 (5%)
Spontaneous start of puberty			
Not yet (at time of counselling)	9 (14%)	5 (14%)	14 (15%)
Yes	19 (29%)	26 (74%)	45 (45%)
No	32 (49%)	4 (11%)	36 (36%)
Missing	5 (8%)	0 (0%)	5 (5%)
Tanner stage (after puberty)			
2	3 (16%)	0 (0%)	3 (7%)
3	2 (11%)	1 (4%)	3 (7%)
4	3 (16%)	3 (12%)	6 (13%)
5	11 (58%)	21 (81%)	32 (71%)
Missing	0 (0%)	1 (4%)	1 (2%)
Spontaneous menarche (after puberty)			
Yes	13 (68%)	26 (100%)	39 (87%)
No	6 (32%)	0 (0%)	6 (13%)
Fertility preservation planned			
None	2 (3%)	16 (46%)	18 (18%)
Ovarian tissue cryopreservation^b^	63 (97%)	10 (29%)	73 (73%)
Oocyte cryopreservation	0 (0%)	9 (26%)	9 (9%)

a Includes isochromosome, ring chromosome, deletions and translocations of X, or Y-fragment.

b One patient who cryopreserved ovarian tissue at ages 12 returned for oocyte cryopreservation at age 16, only counted as ovarian tissue cryopreservation in the table.

Of all patients with Turner karyotype, 39% had monosomal karyotype (45X), 20% had 45X/46XX or 45X/47XXX mosaicism and 36% had an X-chromosome structural anomaly. After 2006 only 20% of the referred girls had a 45X monosomy (**Table 1**).

### Fertility preservation methods applied

Ovarian tissue cryopreservation was planned for 74% of all patients; 89% of the prepubertal girls, 71% of adolescents and 54% of adult women. Oocyte cryopreservation was planned for 10% of adolescents and 15% of adults. In total, 18% of the patients did not undergo any fertility preservation treatment; 11% of girls, 19% of adolescents and 31% of adult women. Two patients who cryopreserved tissue also returned to cryopreserve oocytes later (**Table 2**).

**Table 2:** Ovarian tissue biopsy by year of referral.

	1999-2005 N=63	2006-2021 N=10	All patients N=73
Age group			
Girls (1-12y)	21 (33%)	3 (30%)	24 (33%)
Adolescents (13-17y)	35 (56%)	7 (70%)	42 (58%)
Women (18-27y)	7 (11%)	0 (0%)	7 (10%)
Ovaries			
No abnormalities detected	28 (44%)	9 (90%)	37 (51%)
Streak ovaries	30 (48%)	1 (10%)	31 (42%)
Ovaries absent	5 (8%)	0 (0%)	5 (7%)
Uterus			
No abnormalities detected	53 (84%)	10 (100%)	63 (86%)
Small uterus	2 (3%)	0 (0%)	2 (3%)
Uterus non visualized	8 (13%)	0 (0%)	8 (11%)
Biopsy taken			
Yes	52 (83%)	10 (100%)	62 (85%)
No	11 (17%)	0 (0%)	11 (15%)
Follicles in biopsy			
Not analyzed	6 (10%)	10 (100%)	16 (22%)
No	42 (67%)	0 (0%)	42 (58%)
Yes	15 (23%)	0 (0%)	15 (21%)

Among the 73 patients with Turner karyotype who were planned for ovarian tissue biopsy, no ovarian pathologies were detected for 51% of patients, streak ovaries were found in 42% and 7% had no ovaries (**Table 2**). In a minority of patients, the uterus was either small (3%) or non visualized (11%). In 11 cases, no ovarian tissue could be retrieved due to absent ovaries or streak ovaries observed during the laparoscopic procedure. In total, 85% of the patients had ovarian cortical tissue cryopreserved (**Table 2**).

Between 1999 and 2005, 65 girls were referred for fertility preservation, 57 of them as part of a study to evaluate fertility potential in Turner patients through measurements of fertility markers and through follicle counts in biopsied tissue (3). Among these 65 girls, 29% had spontaneous start of puberty and 97% were planned for ovarian tissue cryopreservation (**Table 1**). Follicles were found in 15 of the 57 biopsies analyzed (26%). In these 15 biopsies, the number of follicles varied from 0.7–1200/mm^3^. All but one patient with streak ovaries did not have observable follicles. Adolescents were more likely to have follicles present (30%) than girls (16%) and adult women (17%) (**Table 2**).

Since 2006, 35 Turner patients have been referred for fertility counselling. 26 (74%) patients had both spontaneously entered puberty and had menarche, 5 (14%) had not yet entered puberty but were below the age of twelve, and 4 (11%) had not entered puberty. Among them 19 (54%) proceeded with fertility preservation measures (**Table 1**). 10 (29%) underwent biopsies for ovarian tissue cryopreservation and 10 (29%) were stimulated for oocyte cryopreservation (**Table 2**). All patients undergoing oocyte cryopreservation had spontaneously entered puberty and also had spontaneous menarche. Among the 16 (46%) that have not yet done any FP measures, five (28%, currently at a mean age of 14) are planned for follow up, five (28%, with a mean age of 17 at counselling) had spontaneous puberty and menarche with hormonal levels indicating remaining fertility but are currently not planned for fertility preservation, six (35%, with a mean age of 19 at counselling) had low hormonal levels and are not planned for fertility preservation, among them one diseased. Among the six with low hormonal levels three had a monosomy.

The 10 patients that proceeded with oocyte cryopreservation, underwent a total of 15 cycles of controlled ovarian stimulation. 8 patients successfully cryopreserved oocytes. The mean age at stimulation was 17.2 years. In the seven patients with AMH measurements, the mean level was 1.1 µg/l. The mean number of mature oocytes cryopreserved in each stimulation cycle was 5.1 (range 0-19) (**Table 3**). The oocytes were cryopreserved using vitrification.

**Table 3:** Oocyte cryopreservation.

	Adolescents and women N=10 **
Age, y	17.2 ± 2.6 (14–22)
AMH, µg/l	1.1 ± 1.0 (0.3-3.2)
Number of stimulations, n	1.5 ± 0.5 (1–2)
Oocytes cryopreserved* per cycle, n	5.1 ± 5.4 (0-19)
Oocytes cryopreserved* per patient, n	7.4 ± 7.0 (0-19)

Data from patients referred for Fertility Preservation (FP) at Karolinska University Hospital between 1998 and 2021, who underwent controlled hormonal stimulation for oocyte cryopreservation. Numbers presented are mean ± SD (range). The stimulation cycles were performed 2011-2022 and all oocytes were cryopreserved using vitrification. AMH, Anti-Müllerian hormone.

*Information on number of oocytes missing for one patient, no oocytes could be cryopreserved for two patients.

** 5 patients underwent repeated treatments.

### Fertility treatments and follow-up

At the end of follow-up, 14 girls and adolescents were below 20 years of age, 41 women were in their 20’s and 42 in their 30’s. Three patients were lost to follow up; one deceased and two emigrated. A total of 26 adolescents and women had documented contact with healthcare concerning their fertility, and 20 returned to the reproductive medicine unit. Eight girls and women returned for further fertility counselling, three women underwent a new fertility preservation and seven women elected fertility treatments using egg donation. To date, six women have given birth following fertility treatment using donated oocytes, whereof two had treatments abroad. Three women have reportedly had children following spontaneous conception; one woman with X-chromosome structural anomaly, one woman with mosaicism and one woman with monosomy. No births following the use of stored oocytes have been achieved so far.

### Ovarian tissue transplantation

Two women in this cohort have undergone re-transplantation of cryopreserved ovarian tissue. In one case the karyotype showed X-chromosome structural anomaly and in the other case a mosaicism. None of the women have regained ovarian functionality as measured by repeated blood samples estimating levels of hormone secretion (Serum Estradiol, AMH, FSH, LH, Progesterone).

## Discussion

This unique cohort provides insight into the fertility choices and options available for young girls with Turner syndrome. In Sweden fertility counselling and fertility preservation is tax-funded and girls and women with Turner syndrome are currently followed up throughout life by multidisciplinary teams at Turner centers established at all university hospitals according to the National Healthcare Program for Turner syndrome. Reproductive options are discussed soon after diagnosis and also at the transition from pediatric to adult healthcare. In presence of any congenital heart or vessel disease pregnancy is not recommended. The current recommendations are that a girl that has a spontaneous start of puberty should be referred for reproductive counselling, and if feasible, based on health and fertility status, stimulated for oocyte cryopreservation before her oocyte reserve is too much reduced by the rapid atresia.

This cohort has been assembled from the fertility preservation unit at Karolinska University Hospital over a period of 22 years. It should be noted that most of the patients who were included before 2006 (57 of 65) underwent ovarian tissue cryopreservation independent of age and pubertal status, as part of a study to evaluate the fertility potential of Turner girls (3). Based on the results from that study, as well as national and international guidelines, most girls included from 2006 and onwards have been referred for fertility preservation only at signs of spontaneous puberty or at expected start of puberty due to a karyotype with mosaicism. When possible, oocyte cryopreservation has been the fertility preservation method of choice, as the method gained recognition as a clinically established method earlier than ovarian tissue cryopreservation, which was still considered experimental until very recently (21).

A majority of the counselled patients underwent fertility preservation, most often through ovarian tissue cryopreservation. Nearly half the patients who underwent a tissue biopsy had observed abnormalities of the ovaries and one in ten patients had no uterus. In total 11 laparoscopies to obtain ovarian biopsies had to be terminated without tissue retrieval. Following the implementation of stricter criteria for referral, all planned biopsies could proceed successfully.

The implementation of more accurate methods of estimating the female ovarian reserve, such as using biochemical markers including serum AMH, and the adherence to updated guidelines drastically reduced the referral rate and only 35 girls and women with Turner syndrome have been counselled between 2006 and 2020. In this later cohort, 74% had spontaneous menarche, ten patients have cryopreserved ovarian tissue, eight patients have undergone successful oocyte cryopreservation, most in late adolescence, and six are planned for follow-up. Only six patients referred after 2006 have been counselled to proceed with other options for family planning due to low hormonal levels or other health issues. This can be compared to the 65 girls counselled between 1999 and 2005 where 71% did not have signs of spontaneous puberty, no ovarian stimulations for oocyte cryopreservation were performed and 97% of patients were planned for ovarian tissue cryopreservation, whereof 15 patients (24%) had observable follicles the in biopsied tissue.

The live birth rate after re-transplantation of cryopreserved ovarian tissue has been estimated to 33-38% in several other patient groups (22–24). However, the potential for pregnancy and live birth is directly correlated with the number of functional primordial follicles available in the biopsied ovarian tissue (25). To date outcome data are limited when ovarian tissue cryopreservation is performed at a very young age (26–28). In patients with Turner syndrome, where rapid atresia is common, the success rate of ovarian tissue re-transplantation is uncertain. While age alone was previously shown as a non-significant factor in predicting the occurrence of follicles in girls above 12 years of age, it should be noted that these results were from a cohort where the patients where all below the age of 20 (3).

Six women in the cohort have given birth following assisted reproduction with donated oocytes. In addition, we have observed five spontaneous pregnancies followed by live births in three women from the cohort (3%), but cannot exclude the possibility of additional undocumented cases. A large French study by Bernard et al. (29) reported a live birth rate of 3.8% (18/480) and an overall 5.6% (27/480) prevalence of spontaneous pregnancies in women with Turner syndrome. Most of these pregnancies occurred in women with mosaic karyotype and only 0.4% (2/480) in women with a non-mosaic (45X) karyotype. Among the three women with children after spontaneous pregnancy in our cohort, one had a monosomy, one an X-chromosome structural anomaly and one a mosaicism. In a recent UK study including 127 pregnancies in 81 women with Turner syndrome (30), 58% (73/127) of pregnancies were spontaneously conceived and all others were conceived using oocyte donation. Among pregnancies in women with monosomy (45X), 29% (9/31) were spontaneously conceived. To our knowledge, there is only one report so far on a successful fertility treatment using cryopreserved oocytes in a woman with Turner syndrome (31).

This study has amassed a large prospective cohort of Turner patients but is limited by the lack of data on follicle status in the biopsies taken after 2006 and incomplete information on spontaneous births. Further, most patients in the cohort are still relatively young. The return rate in the cohort is expected to increase as all patients are still eligible for fertility preservation and some are still prepubertal or adolescent. All patients that have proceeded with fertility treatments can be found among the 42 patients in their thirties. None of the patients that have preserved oocytes have yet reached 30 years of age.

We report two attempts at re-transplantation of ovarian tissue, but as of yet there has been no successful re-transplantation nor any use of the cryopreserved oocytes in our cohort. As the scarcity of promising follow-up data discourages routine use of early ovarian tissue cryopreservation, Turner girls with early onset atresia currently lack promising FP prospects. However, the implementation of the current guidelines with counselling and follow up from onset of puberty has proven useful for identifying Turner girls eligible for fertility preservation.

## Data availability statement

The datasets presented in this article are not readily available because the datasets could possibly identify the individual participants due to the limited cohort size. Requests to access the datasets should be directed to kenny.rodriguez-wallberg@ki.se.

## Ethics statement

The studies involving human participants were reviewed and approved by Ethical Review Board of Karolinska University Hospital (Dnr 427/03) and the Regional Ethics Committee of Stockholm (Dnr 2011/1158-31/2, 2014/470-32, 2016/2530-32 and 2018/2255-32). Written informed consent to participate in this study was provided by the participants’ legal guardian/next of kin.

## Author contributions

Conceptualization, KR-W. Methodology, KR-W and FL. Formal Analysis, FL. Data Curation, KR-W and FL. Writing – Original Draft Preparation, KR-W, HN and FL. Writing – Review & Editing, KR-W, FS, HN, FL. Funding Acquisition, KR-W. All authors contributed to the article and approved the submitted version.
